# Prognostic Factors in Canine Acute Intervertebral Disc Disease

**DOI:** 10.3389/fvets.2020.596059

**Published:** 2020-11-26

**Authors:** Natasha J. Olby, Ronaldo C. da Costa, Jon M. Levine, Veronika M. Stein, The Canine Spinal Cord Injury Consortium (CANSORT SCI)

**Affiliations:** ^1^Department of Clinical Sciences, North Carolina State University College of Veterinary Medicine, Raleigh, NC, United States; ^2^Department of Veterinary Clinical Sciences, College of Veterinary Medicine, The Ohio State University, Columbus, OH, United States; ^3^Department of Small Animal Clinical Sciences, College of Veterinary Medicine and Biomedical Sciences, Texas A&M University, College Station, TX, United States; ^4^Department for Clinical Veterinary Medicine, Vetsuisse Faculty, University of Bern, Bern, Switzerland

**Keywords:** paraplegia, ambulation, dog, pain perception, spinal cord injury, acute intervertebral disc extrusion, acute non-compressive nucleus pulposus extrusion

## Abstract

Knowledge of the prognosis of acute spinal cord injury is critical to provide appropriate information for clients and make the best treatment choices. Acute intervertebral disc extrusions (IVDE) are a common cause of pain and paralysis in dogs with several types of IVDE occurring. Important prognostic considerations are recovery of ambulation, return of urinary and fecal continence, resolution of pain and, on the negative side, development of progressive myelomalacia. Initial injury severity affects prognosis as does type of IVDE, particularly when considering recovery of continence. Overall, loss of deep pain perception signals a worse outcome. When considering Hansen type 1 IVDE, the prognosis is altered by the choice of surgical vs. medical therapy. Concentration of structural proteins in the plasma, as well as inflammatory mediators, creatine kinase, and myelin basic protein in the cerebrospinal fluid (CSF) can provide additional prognostic information. Finally, cross-sectional area and length of T2 hyperintensity and loss of HASTE signal on MRI have been associated with outcome. Future developments in plasma and imaging biomarkers will assist in accurate prognostication and optimization of patient management.

## Introduction

Intervertebral disc disease (IVDD) is a common cause of acute spinal cord injury in dogs, due in large part to the high rates of early disc degeneration in chondrodystrophic breeds (1). Indeed, acute, severe thoracolumbar spinal cord injuries account for ~4% of cases presenting to emergency rooms in North America, with 74% of these cases due to some form of IVDD (2) and ~20,000 surgeries for the condition taking place annually (3). Paralysis is an extremely stressful clinical problem for pet owners, who have to process complex information on the underlying disease, the risks and costs of the procedures needed to diagnose and treat their pet, and the possibility that their pet might suffer permanent deficits or death. Thus, rapid and accurate patient assessment and a realistic portrayal of the clinical picture and prognosis is important for the owner at time of presentation to the veterinarian. Moreover, this information aids the veterinarian with appropriate patient triage and with setting realistic functional recovery goals that will allow early detection when a patient is deviating from an expected course. Indeed, the importance of prognostication to humans with traumatic spinal cord injury is such that multivariable clinical prediction models have been developed for this purpose (4). In addition, as increasing numbers of clinical trials are performed in this clinically diverse population of dogs, identifying prognostic biomarkers that quantify injury severity more accurately than clinical assessment alone can refine patient inclusion criteria or serve as covariates in trials, increasing study power and efficiency.

The prognosis, or “before (from the Greek, pro) knowledge (from the Greek, gignoskein)” of a disease is a forecast of disease course following its onset, in this case, using standard treatment. It refers to the possible outcomes of a disease and the frequency with which these outcomes can be expected to occur. A prognostic factor is a measurement that is associated with clinical outcome in the absence of therapy or with the application of a standard therapy (5). A biomarker is “a defined characteristic that is measured as an indicator of normal biological processes, pathogenic processes, or biological responses to an exposure or intervention, including therapeutic interventions. Molecular, histologic, radiographic, or physiologic characteristics are types of biomarkers” (https://www.ncbi.nlm.nih.gov/books/NBK326791/). Moreover, a prognostic biomarker is “a biomarker used to identify likelihood of a clinical event, disease recurrence or progression in patients who have the disease or medical condition of interest.” In the context of intervertebral disc extrusion (IVDE)-induced spinal cord injury, the clinical events being prognosticated include the *recovery of independent walking* (both speed and level of recovery) and *fecal and urinary continence*. Resolution of pain is also important, and usually considered for certain subsets of IVDH that cause extreme pain such as cervical IVDE. Finally, the chances of *development of progressive myelomalacia*, a complication that is usually fatal, is also prognosticated in Hansen type 1 IVDE specifically.

Over the last few decades, there have been numerous studies evaluating prognostic factors for recovery of ambulation and continence after IVDH-induced thoracolumbar spinal cord injury, fewer after cervical spinal cord injury. Many of these studies consider clinical parameters, but their findings can be contradictory. Quantification of lesion extent using imaging, originally using myelography and more recently, magnetic resonance imaging (MRI), has received a lot of attention. Finally, biomarkers measured within the blood and cerebrospinal fluid (CSF) have been investigated. In this article we will consider prognostic factors for dogs with acute spinal cord injury due to acute IVDH. The majority of published data reports outcomes in thoracolumbar IVDH, but data on cervical IVDH have also been included. We will focus on Hansen type 1 IVDE but have also included prognostic information on acute non-compressive nucleus pulposus extrusions (ANNPE) and FCEM (fibrocartilagenous embolic myelopathy) as well as hydrated nucleus pulposus extrusions (HNPE). Fibrocartilagenous embolism is included both because of its clinical similarity to ANNPE and because it represents a form of intervertebral disc-induced acute spinal cord injury.

When presenting data on prognosis to clients, it is extremely important to educate them on what that data represents for their individual dog and on how to use the data in the recovery period. Clients need accurate information in order to make the best decision initially and perhaps the most difficult concept for them is understanding that the data presented represents the behavior of a population; the precise prediction of outcome for an individual is not possible. Once beyond the initial decisions about treatment, providing clients with a timeline for certain thresholds to be crossed, while again explaining the variability across a population and factors that might influence their individual dog, can ensure that a dog that is not following an expected recovery curve, perhaps due to a comorbidity or a complication of the initial injury, is identified in a timely manner. This discussion can also help clients to understand that decisions can be made at many different stages of injury and recovery, and avoid a decision being made in a time of great stress that might be regretted at a later date.

## Prognostic Factors

### Clinical Presentation

Several different parameters have been evaluated for their prognostic utility including signalment, speed of onset and duration of signs (in particular duration of paralysis), and severity of neurological deficits. The most likely underlying condition is also taken into account because it will affect prognosis. This occurs because different forms of IVDD cause differing types and distribution of spinal cord pathology. The vast majority of these studies are retrospective in nature and variation in patient populations, diagnostic and therapeutic protocols and reporting and methods of follow up make it difficult to draw strong conclusions.

#### Severity of Neurological Deficits

The exception to the limitation of clinical parameters as prognostic factors is severity of neurological deficits, which is the most immediately accessible, simple and reliable prognostic indicator for animals with spinal cord injury. This is true regardless of neuroanatomic location and is equally true in human spinal cord injury (6). Given the poor regenerative capabilities of the adult CNS, it is not surprising that gauging the extent of permanent tissue loss is extremely important to establish a prognosis. It is common practice to assign animals with thoracolumbar spinal cord injury to one of six categories based on the severity of their clinical signs in a scale known widely as the Modified Frankel Scale (MFS) (7, 8). The frequency of signs within each category in this scale has been estimated (Table 1) (9). There are numerous slightly different variations on this scale in the veterinary literature, with numbers assigned in different directions and subcategories developed. This makes comparisons between studies confusing and so for the purposes of accurate reporting in this paper, clinical severity has been categorized using description of the signs (Table 1).

**Table 1:** Definition of categories of neurologic injury commonly used clinically.

**Description**	**MFS Scale 1 (7)**	**MFS Scale 2 (8)**	**Frequency in reported studies (9)**	**ASIA impairment scale (6)**
Normal	0	6	NA	E
Painful, no neurological deficits	1	5	NA	
Ambulatory paraparetic/ataxic	2	4	30%	D
Non-ambulatory paraparetic	3	3	22%	C
Paraplegic intact DPP *Might be subcategorized by:* • *Presence of pain in both hind feet and tail vs. only one location* • *Presence of superficial pain* • *Presence of voluntary urination*	4	2 (superficial and deep pain present) 1 (lacks superficial pain)	30%	B B
Paraplegic NDPP	5	0	16%	A

*Note numbers are assigned using opposite conventions by different authors. These categories correspond well to the human American Spinal Injury Association (ASIA) scale (6). In this article the description of each category is used to avoid confusion when interpreting a wide range of literature. MFS, Modified Frankel Scale; NA, not applicable—data derived from studies of dogs presenting for surgery; DPP, deep pain perception; NDPP, no deep pain perception*.

#### Evaluation of Pain Perception

Prognosis for recovery of independent ambulation is influenced by the *presence of pain perception* (10–12). Not surprisingly for such an important clinical variable, there is a range of different terminology used in the literature. Historically the term “deep pain perception” (DPP) has been used—referring to the response to an extremely noxious stimulus applied over the bone of a digit. More recently, the term deep has been omitted and authors use the terms pain perception and nociception. Sometimes “deep nociception” appears. In this article we use the term DPP when discussing prognostic indicators because it is familiar to most veterinarians and conveys the importance of applying a strong noxious stimulus when determining the presence of pain perception.

Because of its clinical implications, assessment of pain perception should be made extremely carefully in any animal that lacks motor function. This is performed ideally in a calm animal, using an instrument with relatively wide jaws such as needle drivers or pliers (to avoid cutting the skin as pressure is applied). While placing an animal on its side to perform this test allows a clear view of the response, if they are fighting to get up, it can be difficult to interpret their behavior. If this occurs, the animal should be placed in whatever position allows clear access to the limb being tested with the animal resting quietly. Pressure is applied over the digit being tested and a gentle squeeze is applied to produce a withdrawal reflex (if present) and then pressure is increased until the patient demonstrates perception of the stimulus such as vocalization, looking around, or moving away (Supplementary Video 1). In animals with blunted perception, the response might be as subtle as an alteration in breathing pattern or dilation of pupils. Any repeatable behavioral indication that the animal can feel the stimulus is taken as DPP being present. Both medial and lateral digits should be tested in each foot and the base of the tail should be tested (using the handles of the forceps). Presence of pain perception in any one of these locations places the animal in the prognostic category of having DPP (10, 13).

#### Recovery in Animals With Intact DPP

The prognosis for recovery of independent ambulation and continence in animals that have intact DPP, even if apparently blunted, is good to excellent depending on the treatment pursued (Table 2) and the type of disc herniation that occurred (Table 3). The speed of that recovery is influenced by the severity of motor impairment at presentation, altering prognosis for walking at 2, 4–6, and 12 weeks (Table 2). These benchmarks are extremely useful to indicate when a dog might not be recovering as expected, triggering a timely re-evaluation by the veterinarian. Recovery of fecal and urinary continence in dogs with DPP due to Hansen Type 1 IVDE matches recovery of walking. However, persistent fecal and urinary incontinence have both been reported in animals with incomplete injuries due to ANNPE and FCEM in spite of recovery of ambulation (Table 3).

**Table 2:** Summary of prognosis for acute TL-IVDE based on presenting grade of injury and treatment choice.

**Grade**	**Overall recovery with conservative management (%)**	**Overall recovery with surgery (%)**	**Recovery at 2 w (%)**	**Recovery at 4–6 w (%)**	**Recovery at 3 m (%)**	**Development of PMM (%)**
Ambulatory paraparetic	72.5 (*n* = 116)	98.4 (*n* = 318)	84	92	93.9	0
Non-ambulatory paraparetic	79.8 (*n* = 74)	93 (*n* = 341)	77.8	88.9	92.8	0.6
Paraplegic with DPP	56 (*n* = 77)	93 (*n* = 548)	70.8	78.2	83.2	2.7
Paraplegic NDPP	22.4% (*n* = 48)	61 (*n* = 502)	26.5	42.3	53.8	13.9

*w, weeks; m, months; PMM, progressive myelomalacia; NDPP, no deep pain perception; DPP, deep pain perception (10, 12–21)*.

**Table 3:** Outcomes of dogs with different types of acute thoracolumbar intervertebral disc disease.

**Grade**		**Ambulatory paraparetic**	**Non-ambulatory paraparetic**	**Paraplegic with DPP**	**Paraplegic with NDPP**
ANNPE (22, 23)	Amb	100% (*n* = 84)	100% (*n* = 105)	100% (*n* = 40)	Unknown
	UC	96.9% (*n* = 65) 100% (*n* = 19)	91.1% (*n* = 90) 100% (*n* = 15)	82.1% (*n* = 28) 92% (*n* = 12)	Unknown
	FC	92.3% (*n* = 65) 100% (*n* = 19)	75.7% (*n* = 90) 93% (*n* = 15)	46.4% (*n* = 28) 58% (*n* = 12)	Unknown
FCEM/	Amb		87.5% (*n* = 301)		43% (*n* = 7)
ischemic myelopathy	UC		99% (*n* = 40)		Unknown
(24–26)			70.4% (*n* = 51)		
	FC		97% (*n* = 40)		Unknown
			59.3% (*n* = 51)		

*ANNPE, acute non-compressive nucleus pulposus extrusion; FCEM, fibrocartilagenous embolic myelopathy; Amb, ambulatory; UC, urinary continence; FC, fecal continence; NDPP, no deep pain perception; DPP, deep pain perception*.

Recovery of independent ambulation and resolution of pain in dogs with cervical IVDE has also been reported with and without spinal cord decompression and in general is excellent with surgery (Table 4). However, potentially serious complications of hemorrhage, hypoventilation and bradycardia, vertebral subluxation and aspiration pneumonia have all been reported and the development of an adverse event of this manner does worsen prognosis (28, 42–45). The majority of dogs with hydrated nucleus pulposus extrusions (HNPE) present with cervical extrusions. These dogs have an excellent prognosis for recovery with or without surgery, even in the presence of respiratory compromise (Table 4).

**Table 4:** Outcomes of dogs with different types of acute cervical intervertebral disc disease.

	**Conservative**	**Surgery**
Hansen type 1 IVDE	72.8% (*n* = 197)	96.2% (*n* = 786)
(27–31)		
HNPE (32–37)	98.5% (*n* = 45)	95.5% (*n* = 97)
ANNPE (38)	100% (*n* = 12)	
FCEM (39–41)	82.7% (*n* = 39)	

*A successful outcome is recovery of ambulation and resolution of cervical pain. All severities of injury are considered together because studies have shown no effect of injury severity on outcome. Persistent cervical pain is the most common reason for treatment failure for Hansen type 1 IVDE. ANNPE, acute non-compressive nucleus pulposus extrusion; FCEM, fibrocartilaginous embolic myelopathy*.

#### Recovery in Animals With No DPP

The prognosis for animals that lack DPP is less certain, with recovery rates for independent walking in dogs with surgically managed thoracolumbar IVDE ranging from 30 to 75% in different studies (11–16). Overall, ~60% of dogs with Hansen type 1 IVDE recover DPP and ambulation by 6 months after injury (Table 2). The timing of recovery of pain perception is important, because once it is present, the prognosis for recovery of ambulation is excellent. One study found that 62% of dogs that did recover DPP recovered it within 4 weeks, another 30% by 12 weeks and one dog (8%) recovered it at 36 weeks (16). The prognosis for recovery of fecal and urinary continence in these dogs is not quite the same as recovery of independent walking. In dogs with Hansen type 1 IVDE that do recover DPP and walking, ~40% do not recover normal fecal continence and 30–53% do not recover normal urinary continence (13, 16). While in the majority of cases, owners find the level of continence acceptable, it is important to note it might not be normal and accidents will be more likely to happen than prior to injury.

The prognosis for recovery in dogs with ANNPE and FCEM that present with paraplegia without DPP is considered poor and the majority of these dogs are euthanized within a week of injury. As such, these cases are scarcely reported in the literature and it is extremely difficult to establish what their prognosis would be if managed long term. There is a recent report of three dogs with ANNPE that recovered walking, but none recovered fecal continence and 2/3 remained urinary incontinent (22). A review of the literature on FCEM revealed seven dogs in this category for which long term outcomes were available; three of the seven recovered ambulation but there is no information on their continence (24).

The prognosis of dogs with cervical lesions that lack DPP is difficult to report because so few dogs present with this severity of injury (29). This reflects the high mortality rate due to hypoventilation and brady-arrhythmias in dogs with functionally complete cervical spinal cord injury (46).

There are special considerations in dogs that lack DPP when discussing prognosis. The first is the development of progressive myelomalacia (PMM) and the second is the prognosis for recovery of ambulation if DPP is not recovered. Progressive myelomalacia is a very important consideration due to the gravity of this condition, and the most important risk factor for development of this condition is injury severity (47, 48) (Table 2). Indeed between 9 and 33% of DPP negative dogs with Hansen type 1 IVDE can develop this condition, with most studies reporting a rate between 9 and 17.5%, and one study reporting a rate of 33% in French bulldogs specifically (11, 14–16, 47, 48). It is extremely important to warn owners of the potential development of PMM in paraplegic DPP negative dogs and to monitor for it both pre and post-operatively (49).

The prognosis of dogs developing independent walking without recovery of DPP has also been investigated in dogs that have suffered Hansen type 1 IVDE. Two studies have reported this specifically and in total 27/88 (31%) dogs that did not regain DPP recovered the ability to walk. In both studies the median time to walking was 9 months with a range of 2–28 months (13, 16). It is important to note that while these dogs did regain some ability to urinate and defecate, none had normal continence. The prognosis for recovery of walking in this population of dogs has been reported to be improved to a recovery rate of 59% with intensive physical rehabilitation (50).

#### Spinal Shock and Schiff Sherrington Posture

The presence of spinal shock and Schiff Sherrington posture at presentation have been described in dogs (9, 51). Spinal shock occurs much more frequently with FCEM and ANNPE due to the peracute onset of signs in these conditions (52). The prognostic significance of spinal shock has been evaluated and its presence is associated with the development of fecal incontinence in ANNPE but does not appear to affect recovery of ambulation (22, 52). The prognostic significance of the Schiff Sherrington posture has not been evaluated. The assumption of its importance is likely because it is easily recognized in severe thoracolumbar SCI cases, but the presence or lack thereof of DPP should be used as the indicator of prognosis in these cases, as previously discussed.

#### Signalment

Breed and age affect the likelihood of a particular type, location and severity of IVDD. For example, younger dogs with acute TL IVDE present with more severe neurological signs (53) and acute TL IVDE occurs at a younger age, a more caudal site and a greater severity of neurological deficits in French bulldogs when compared with Dachshunds. As a result of the severity and tendency for a more caudal, lumbar location of their spinal cord injury, French bulldogs are more likely to develop PMM with rates as high as 33% in deep pain negative dogs (11). However, in these examples, the prognostic factors at presentation are severity of signs and location of disc extrusion.

Breed has been evaluated as a prognostic factor in several studies, but analysis is somewhat hampered by the overwhelming prevalence of Dachshunds (Table 5). Regardless, no study has found an effect of breed on prognosis. Similarly, sex does not alter prognosis (12, 15, 47). Several studies have evaluated the effect of age on prognosis and results have been somewhat contradictory, with one study showing increased age slows the speed of recovery in acute TL IVDE, but does not alter the final outcome, another suggesting it reduces the final recovery level, while others show no effect on final recovery (12, 16). A clear conclusion on the role of age as a prognostic factor cannot be drawn. Body weight has been investigated and results are similarly conflicting. One study on TL IVDE found increased body weight slowed speed of recovery and another found that dogs that weighed >20 kg had a worse outcome. Other studies found no effect of body weight on final outcome (12, 16, 47, 54–56). By contrast, when evaluating a population of non-ambulatory tetraparetic dogs, small breed dogs are six times more likely to have a successful recovery than large breeds (30).

**Table 5:** Summary of studies evaluating the relationship between signalment and prognosis.

	**Study**	**Population of dogs**	**Conclusion**
Breed	Ruddle et al. (12) retrospective	*N* = 250 all non-ambulatory with and without pain perception	No effect of breed on recovery speed or final outcome
	Castel et al. (47) retrospective	*N* = 197, all deep pain negative	No effect of breed on final outcome. Recovery speed not examined
Age	Ruddle et al. (12) retrospective	*N* = 250 all non-ambulatory with and without pain perception	No effect of age on recovery speed, increasing age worsens outcome
	Olby et al. (16) retrospective	*N* = 64, all deep pain negative	Increased age slows speed of recovery but not final outcome
	Davis and Brown (54) retrospective	*N* = 112, all non-ambulatory with pain perception	No effect of age on recovery speed or final outcome
	Jeffery et al. (15) prospective	*N* = 78, all deep pain negative	Age has no effect on final outcome. Recovery speed not examined
	Castel et al. (47) retrospective	*N* = 197, all deep pain negative	Age has no effect on final outcome. Recovery speed not examined
Weight	Ruddle et al. (12) retrospective	*N* = 250 all non-ambulatory with and without pain perception	Weight has no effect on speed of recovery or final outcome
	Olby et al. (16) retrospective	*N* = 64, all deep pain negative	Increased weight slows speed of recovery but not final outcome
	Davis and Brown (54) retrospective	*N* = 112, all non-ambulatory with pain perception	Weight has no effect on speed of recovery or final outcome
	Macias et al. (55). Retrospective, dogs >20 kg	*N* = 63, all severities of injury	Weight has no effect on final outcome when compared to other studies
	Bull et al. (56) retrospective	*N* = 238, all severities of injury, cervical and TL	Dogs >20, g had a worse outcome than dogs <20 kg
	Shaw et al. (57) retrospective	*N* = 121, split T3-L3 and L4-S3. ANNPE, HNPE, FCE and Hansen type 1 IVDE	Dogs weighing >15 kg had a worse outcome than dogs <15 kg
	Hillman et al. (30) retrospective	*N* = 32, cervical IVDE, all non-ambulatory	Dogs weighing <15 kg are 6 × more likely to recover completely than dogs weighing >15 kg
	Cherrone et al. (29) retrospective	*N* = 190, cervical IVDE, small vs. large breed, all severities of injury	Large breed dogs more likely to have a recurrence. No effect on speed of recovery or final outcome

*Unless noted specifically, all studies evaluated dogs with TL-IVDE treated with decompressive surgery*.

#### Onset and Duration of Signs

Various studies have evaluated the speed of onset and the duration of signs, in particular the duration from onset of non-ambulatory status to surgical decompression (Table 6). These studies are necessarily hampered by reliance on owner observations and periods during which pet dogs are not observed. In addition, the definition of the times can differ between studies with varying definition of onset (onset of ataxia, vs. pain for example), time to presentation or time to surgery as well as different populations of dog being examined. Large case cohorts are presented in Table 6. Overall, there is no consensus on an effect of speed of onset of signs or duration of signs on overall outcome, but there is some evidence that duration of signs might influence the speed of recovery. There is also some evidence that an interval of >12 h between onset of non-ambulatory status and surgical decompression increases the risk of PMM (47). Finally, there is evidence that delaying surgery until the day following presentation increases the risk of clinical deterioration from which the dog might not recover (59).

**Table 6:** Summary of studies evaluating the relationship between speed of onset and duration of non-ambulatory status.

**Parameter**	**Study**	**Population of dogs**	**Conclusion**
Speed of onset	Scott et al. (14) retrospective	*N* = 34, all deep pain negative	Peracute onset (<1 h) has a negative effect on outcome. Speed of recovery not examined
	Ferreira et al. (58) retrospective	*N* = 71, all paraplegic deep pain positive	Peracute onset (<2 h) has a negative effect on outcome but not speed of recovery
	Olby et al. (16) retrospective	*N* = 64, all deep pain negative	No effect on final outcome. Speed of recovery not examined
	Jeffery et al. (15) prospective	*N* = 78 all without pain perception	No effect on final outcome. Speed of recovery not examined
	Castel et al. (47) retrospective	*N* = 197, all deep pain negative	No effect on final outcome. Speed of recovery not examined
Duration of non-ambulatory status	Scott et al. (14) retrospective	*N* = 34, all deep pain negative	No effect on final outcome or speed of recovery
	Ferreira et al. (58) retrospective	*N* = 71, all paraplegic deep pain positive	No effect on final outcome but duration of paralysis >6 days slows speed of recovery
	Davis and Brown (54) retrospective	*N* = 112, all non-ambulatory with pain perception	Increased duration of paralysis increased speed of recovery, no effect on final outcome
	Olby et al. (16) retrospective	*N* = 64, all deep pain negative	No effect on final outcome. Speed of recovery not examined
	Jeffery et al. (15) prospective	*N* = 78, all deep pain negative	No effect on final outcome. Speed of recovery not examined
	Castel et al. (47) retrospective	*N* = 197, all deep pain negative	No effect on final outcome. Speed of recovery not examined. >12 h duration, increased risk of PMM

#### Location of Intervertebral Disc Extrusion

Several studies have compared outcome in dogs with Hansen type 1 IVDE and found no difference in dogs with T3–L3 vs. L4–S3 localization (Table 7). One study found a worse prognosis in dogs with lower motor neuron (LMN) signs of incontinence (57). Perhaps the most important prognostic detail in this category is the increased risk of development of PMM with discs located in the caudal lumbar vertebrae in dogs that are paraplegic with no pain perception (47, 48). When considering cervical IVDE, adverse events are more likely to occur with disc herniations at C7/T1 (28).

**Table 7:** Prognostic factors associated with location of herniated intervertebral disc.

**Parameter**	**Study**	**Population of dogs**	**Conclusion**
Location	Ruddle et al. (12) retrospective	*N* = 250, all non-ambulatory TL IVDE with and without pain perception	Location of disc herniation has no effect on outcome
	Cardy et al. (60) retrospective	*N* = 162, split between dachshunds and Cocker spaniels, all severities.	Caudal lumbar discs associated with less severe signs and therefore better outcome
	Shaw et al. (57) retrospective	*N* = 121, split T3–L3 and L4–S3. ANNPE, HNPE, FCE and Hansen type 1 IVDE	L4–S3 associated with worse outcome in terms of continence
	Castel et al. (47) retrospective	*N* = 197, all deep pain negative split T3–L3 and L4–S3.	No effect on recovery of ambulation

### Blood and Cerebrospinal Fluid Biomarkers

While the prognosis for recovery can be established quite well in dogs with DPP prior to diagnostics and treatment, those that lack DPP pose a greater challenge. These dogs dichotomize into a group that shows a recovery comparable to those with incomplete lesions and a group in which there is no or limited recovery. These data show us that there is a profound floor effect when evaluating paraplegic DPP negative dogs and that this group includes dogs that have permanent interruption of conduction and those in which that interruption was temporary, perhaps representing conduction block due to edema, energy failure, etc. As a result, there have been many attempts to identify biomarkers that will allow differentiation of these dogs. Such attempts are plagued by the challenges of the influence of duration of injury and the ability to measure them at the time of presentation, to establish meaningful prognoses for owners. None have yet reached the point of clinical utility.

A plasma or serum biomarker would be ideal because it would allow prognostication prior to embarking on expensive advanced diagnostics, but they are somewhat removed from the central nervous system (CNS) compartment and so are likely to require very sensitive measuring techniques. Biomarkers that show the most promise are CNS structural proteins, glial fibrillary acidic protein (GFAP) and phosphorylated neurofilament heavy chain (pNfH) (Table 8) (66–68). Both can be measured in the plasma and serum using ELISA and provide some insight into the severity of CNS injury. Of the two, GFAP is the most discriminating at time of injury, predicting both recovery of ambulation and development of PMM with an accuracy of >80%. Serum pNfH concentrations are more variable at the time of injury and S100β was found to be less useful. Unfortunately, at this time there is no rapid point of care test available, but they have proven useful as a covariate of injury severity in clinical trials (69).

**Table 8:** Summary of studies evaluating the relationship between biomarkers and outcome.

**Biomarker**	**Study**	**Population studied**	**Findings**
CMC CSF MBP and CK	Levine et al. (61)	*N* = 54, all non-ambulatory	CSF MBP <3 ng/mL and CK <38 U/L highly predictive for recovery
	Witsberger et al. (62)	*N* = 54, all non-ambulatory	
CMC CSF cytology	Srugo et al. (63)	*N* = 54, all non-ambulatory	% macrophages and macrophage/mononuclear ratio have high sensitivity and specificity for recovery
	Witsberger et al. (62)	*N* = 54, all non-ambulatory	Cytology has no relationship to outcome
CMC CSF tau	Roerig et al. (64)	*N* = 51, TL and Ce	CSF concentration has high specificity and sensitivity for recovery
CMC CSF inflammatory mediators	Taylor et al. (65)	*N* = 39, all non-ambulatory	CSF concentration of MCP-1 is negatively associated with outcome
Serum pNfH	Nishida et al. (66)	Paraplegic with (*n* = 22) and without deep pain (*n* = 38)	Serum concentration has high specificity but low sensitivity for recovery. Elevated in dogs with PMM
	Olby et al. (67)	*N* = 31, paraplegic without deep pain	Serum concentrations at time of presentation were not associated with recovery
Serum GFAP	Sato et al. (68)	*N* = 51, non-ambulatory	Presence has high sensitivity and specificity for recovery and for PMM
	Olby et al. (67)	*N* = 31, paraplegic without deep pain	
Serum S100beta	Olby et al. (67)	*N* = 31, paraplegic without deep pain	Serum concentrations at time of presentation were not associated with recovery

Evaluation of CSF biomarkers takes the clinician closer to the CNS, but as a result such tests are more invasive and require general anesthesia. Markers evaluated include cytology, myelin basic protein (MBP) with or without creatine kinase (CK), tau, glutamate, matrix metalloproteinase-9 (MMP-9) and inflammatory cytokines (61–65). Some studies have conflicting results, but all are summarized in Table 8 and MBP and CK CSF concentrations in particular are both highly predictive of return of ambulatory function.

### Imaging and Prognosis IVDD

#### Myelography

The first imaging modality used to prognosticate cases of IVDE was myelography. It was based on the extension of an intramedullary pattern, which was interpreted as indirect evidence of the severity and extent of spinal cord swelling (70). Spinal cord swelling, calculated as the ratio of the length of the loss of the dorsal and ventral contrast columns to the second lumbar vertebra (spinal cord swelling: L2 ratio), was correlated with a poor prognosis when it was found to be five or more vertebral bodies. However, a subsequent study could not confirm these findings (14). In the latter study, the ratio for dogs with a successful outcome was 1.7, compared to 2.0 for those with an unsuccessful outcome. Only two dogs had intramedullary pattern longer than five bodies and both dogs recovered.

Myelographic studies of dogs with acute non-compressive nucleus pulposus extrusion (ANNPE) demonstrated an intramedullary pattern and an additional extradural pattern was seen in approximately half of the dogs. The degree of spinal cord swelling was not associated with severity of clinical signs or outcome (71).

An extensive intramedullary pattern with evidence of contrast medium infiltration into the spinal cord has been reported as an indication of progressive myelomalacia (PMM) (72). Infiltration of contrast within the spinal cord parenchyma, however, is not pathognomonic for PMM since it can also be iatrogenic or represent other intramedullary lesions such as syringohydromyelia (72, 73).

#### Magnetic Resonance Imaging

The utility of MRI for diagnostic purposes has been very well-defined and characterized. Its ability to serve as a biomarker is not as clear, although several studies have proposed imaging markers as prognostic indicators. As MRI is routinely acquired as part of diagnostic work-up of IVDE cases, the identification of reliable imaging markers identified on MRI would be invaluable for clinicians and owners.

**Figure 1:**
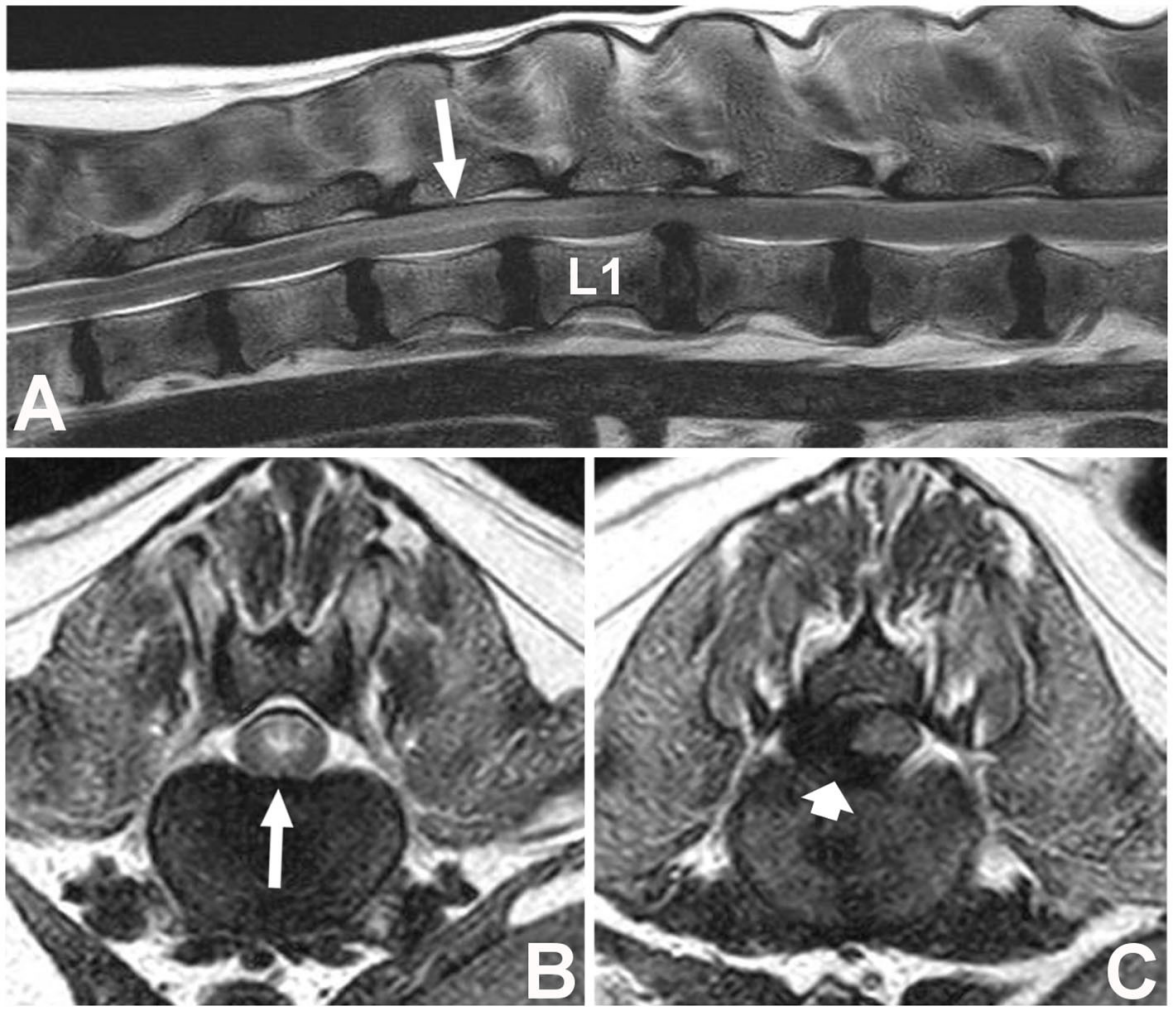
Images of a female spayed, 6-year-old, mixed breed dog with an acute onset of non-ambulatory paraparesis and spinal pain **(A)**. Sagittal T2W image showing moderate ventral spinal cord compression secondary to intervertebral disc extrusion at L1–L2, with associated spinal cord hyperintensity spanning over T13 to L1 (long arrow) **(B)**. Transverse T2W image at T13–L1 showing spinal cord hyperintensity (long arrow) cranial to the compressive lesion **(C)**. Transverse T2 image showing lateralized spinal cord compression caused by a hypointense material between L1–2 (short arrow) found to be extruded disc material at surgery. L1 vertebral body is labeled (L1).

Spinal cord hyperintensity on T2W images has been the most widely investigated parameter (Figure 1). This spinal cord (SC) hyperintensity identified on T2W images has been associated with necrosis, myelomalacia, intramedullary hemorrhage, inflammation, and edema (74–76). Without differentiating the pathologic process more specifically, T2 hyperintensity has been shown to correlate well with the severity of neurologic signs at presentation in dogs with IVDE (53, 77, 78). Its utility as a prognostic indicator is less clear. Even though the first report of the utility of spinal cord hyperintensity indicated that extension of the area of T2W spinal cord hyperintensity on low-field MRI was a reliable predictor of outcome, even more reliable than the absence of deep pain perception (79), these findings could not be reproduced in other studies, primarily those using high-field MRI (Table 9). Use of high-field MRI leads to increase in signal-to noise ratio and consequently to a change in image resolution; therefore, mild intramedullary hyperintensities in sagittal T2W sequences may be more frequently evident using high field magnetic fields compared to low field ones.

**Table 9:** Association of spinal cord hyperintensity detected on T2 weighted MRI in dogs with intervertebral disc extrusion and outcome.

**Study**	**Design and population**	**Magnet strength**	**MR imaging changes**	**Conclusion**	**Comments**
Ito et al. (79)	Retrospective 77 dogs Dogs paraplegic Blinded study Morphologic and morphometric	0.3 T	Only dogs with T2W SC hyperintensity equal to L2 vertebra	Hyperintensity better predictor of outcome than absence of DPP	Large variation between onset signs and MRI—median 7d
Levine et al. (53)	Retrospective 129 dogs with follow-up Ambulatory and non-ambulatory dogs Blinded study Morphologic and morphometric	1.0 T for the majority of cases	Any degree of T2W SC hyperintensity	Direct association between the length ratio of hyperintensity with long-term functional outcome	The large variation in injury severity makes comparison with other studies challenging
Boekhoff et al. (80)	Retrospective 63 dogs All dogs paraplegic Blinded study Morphologic and morphometric	1.0 T	SC T2W hyperintensities ranging from half of L2 vertebra to >2 times L2	Association between extent of T2 hyperintensity with delayed ambulation, not statistically significant	Large variation between onset signs and MRI−62% between 2 and 7 days
Wang-Leandro et al. (81)	Prospective 35 dogs All dogs paraplegic Blinded study Morphologic and morphometric	3.0 T	SC T2W hyperintensities assessed in sagittal plane using L2 vertebra as reference	Length of SC T2 hyper-intensity had no association with motor functional recovery	Only 2 dogs were enrolled with >7 days of onset of signs
Otamendi et al. (82)	Retrospective 47 dogs	3.0 T	SC T2W hyperintensities assessed in sagittal plane using L2 vertebra as reference	No association between T2 hyperintensity and recovery of motor function or PMM	Abstract only

*SC, spinal cord; PMM, progressive myelomalacia*.

Identification of MRI features suggestive of progressive myelomalacia is crucial for prognostic purposes since its identification indicates an abysmal prognosis. The length of spinal cord T2W hyperintensity and the length of intramedullary pattern reflected as loss of CSF signal on HASTE/MR myelography sequences have been used and are presented on Table 10. A recent 3T MRI study proposed that intramedullary hypointensity on T2W images was associated with PMM (86).

**Table 10:** Association of MRI abnormalities associated with progressive myelomalacia (PMM) in dogs with intervertebral disc extrusion.

**Study**	**Design and population**	**Magnet strength**	**MR imaging changes**	**Conclusion**	**Comments**
Okada et al. (83)	Retrospective 12 dogs; five confirmed, seven presumptive Time between onset signs and MRI: median 3.7 d	Low field (0.4 and 0.5 T) for 11/12 dogs 1.5 T for one dog	Length of T2W SC hyperintensity equal L2 vertebra ranged from 6 to 20 times L2	Hyperintensity longer than six times body of L2 characteristic of PMM	Small sample size and only five dogs confirmed
Gilmour et al. (84)	Retrospective five dogs with PMM—necropsy confirmed Time between onset signs and MRI–median 2 ds	1.5 T	T2W length SC hyperintensity/L2: 2.3 and 1.2 (mean, median) Loss of CSF signal ≥ L2 (ratio CSF:L2) on HASTE equal to 10.7 and 8.9 (mean and median)	A ratio of 7.4 of loss of CSF signal ≥ L2 on HASTE had a sensitivity of 100% and specificity of 75%.	Small sample size
Castel et al. (85)	Retrospective 20 dogs with PMM and MRI Number necropsy confirmed not reported Time frame between onset signs and MRI not reported	1.5T	T2W SC hyperintensity longer than six times L2 seen in 45% dogs Loss of CSF signal ≥ 7.4 × L2 (ratio CSF:L2) on HASTE seen in 85% dogs	Loss of CSF signal equal of longer 7.4 × L2 more reliable than T2 hyperintensity	The three dogs with a ratio CSF: L2 HASTE <7.4 were imaged within 12–24 h following onset paraplegia
Balducci et al. (48)	Retrospective 13 dogs with MRI–none necropsy confirmed	0.2 T	-T2W SC hyperintensity longer than 4.57 times L2 seen in 84.6% dogs	Dogs with hyperintensity > 4.57 times L2 were 17.2 times more likely to develop PMM	Dogs may not show T2 SC hyperintensity when imaged <24 h after onset of paraplegia and still develop PMM

High field MRI changes have been associated with outcome in dogs with ANNPE (22, 38) and FCEM (39). A larger lesion on transverse images, quantified as a greater percentage cross sectional area of the spinal cord, has been considered the most useful MRI variable to predict the short- and long-term outcome of dogs with ANNPE and FCEM (22, 38, 39). In ANNPE, dogs with a smaller lesion had a shorter interval to unassisted ambulation. In contrast, a percentage cross sectional area that equals or exceeds 90% of the spinal cord had a 92% chance of having an unsuccessful long-term outcome (38), and a lesion exceeding 40% of the transverse area has been associated with an increased likelihood of long-term urinary and fecal incontinence (22). However, a low-field MRI study of 21 dogs with ANNPE found no association between any MRI parameter with outcome (71).

Advanced MRI techniques have been proposed to increase reliability of MRI as biomarker. A recent study proposed a semi-automated assessment of SC signal changes aiming to minimize interobserver variability (86). Diffusion tensor imaging (DTI) has been used in dogs with naturally occurring spinal cord injury secondary to IVDE (81, 87–89). The spinal cord, primarily white matter, microstructural changes are captured through quantification using DTI techniques. As such DTI is able to detect abnormal SC areas that appear macroscopically normal on T2W sequences. Specific DTI parameters, including tractography, have the potential to serve as prognostic biomarkers, although no specific parameter has been identified (81, 87–89). More information regarding DTI in IVDE can be found in a companion article entitled Diagnostic imaging in intervertebral disc disease.

### Electrophysiological Testing

Attempts have been made to use electrophysiological testing to quantify injury severity and predict prognosis, both at the time of injury and in chronically paralyzed dogs. The majority of this work has been completed in dogs with thoracolumbar IVDE. Two different approaches have been used. The first is to evaluate the descending pathways using magnetic stimulation of the motor cortex (90) and the second is to evaluate the ascending pathways within the spinal cord and projecting to the brain using somatosensory evoked potentials (SSEP) (91).

#### Motor Evoked Potentials

Transcranial magnetic motor evoked potentials (TMMEP) can be elicited reliably in dogs under sedation (92, 93). However, they are extremely sensitive to spinal cord injury and are lost completely in dogs that are paraplegic (94, 95). With less severe injuries, latency increases and amplitude decreases, but these values do not discriminate initial severity as well as clinical assessment and evaluation of MEPs at time of presentation does not provide prognostic information (94, 95). There has been interest in the utility of repeated TMMEP evaluation in dogs that were paraplegic at presentation. Dogs that show recovery of pain perception and motor function recover TMMEPs, leading to the suggestion that this tool can be used to complement assessment of recovery (96). Two groups have evaluated the presence and latency of TMMEPs in dogs that do not regain deep pain perception, and reached different conclusions with one group finding an association between TMMEP presence and recovery or walking, and the other failing to find this association (97, 98). At this time, there is no evidence that evaluation of TMMEP at time of injury can provide prognostic information in acute spinal cord injury due to IVDE.

#### Somatosensory Evoked Potentials

Somatosensory evoked potentials can be elicited by stimulation of a peripheral nerve in a pelvic or thoracic limb (99). Needles are introduced percutaneously to the level of the interarcuate ligament to record from different levels of the spinal cord and subcutaneously to record over the sensory cortex. Various parameters can be recorded including presence or absence of a waveform, latency, amplitude and duration of the potential at the level of the sensory cortex, the conduction velocity of ascending volleys along the spinal cord, particularly across a lesion, the presence and location of conduction block, and the presence and amplitude of an injury potential (100–103). Early work suggested that lack of recordable cortical evoked potential was associated with failure to recover ambulation in dogs with TL IVDE (100). Another study evaluating conduction velocity and amplitude of spinal evoked potentials recorded at T10/11 found that a ratio of conduction velocity to amplitude was predictive of outcome (101). Later studies did not come to the same conclusions, and combined various parameters to discriminate initial injury severity to the level of clinical evaluation (102). The location of conduction block can be evaluated using the evoked injury potential and the distance between conduction block and site of compression might contribute useful information but this has not been investigated further (103). SSEPs have also been evaluated in chronically deep pain negative dogs, but results are conflicting and are not of prognostic utility (97, 98). Currently, there is no clear evidence that prognosis can be established in acute IVDE using SSEPs.

## Conclusions

Understanding the prognosis of spinal cord injury secondary to IVDE is important for client education, optimal patient management and clinical trial design and execution. The prognosis varies with type of IVDE and is influenced by treatment choices. Severity of initial clinical presenting signs is the most useful guide to prognosis at the time of presentation. Biomarkers within the blood, CSF and on imaging can also help to predict outcome. Understanding time to recovery and differentiating between motor and autonomic recovery (continence) can provide invaluable information for veterinarians as they manage dogs that have suffered a spinal cord injury. The manner in which these data are presented and discussed with the owner is a critical part of patient care and it is vital both that accurate information is provided and that owners understand the nature of that information as it relates to their dog. As we move into an era with increased availability of bedside tests of injury severity, and improved accuracy of imaging prognostication, it is likely that patient stratification will improve our ability to perform well-designed clinical trials and to optimize patient care on an individual basis.

## Author Contributions

NO, RdC, JL, VS, and CANSORT SCI contributed to conception of the study. NO, RdC, JL, and VS designed the study. NO wrote the first draft. RdC wrote a subsection. All authors reviewed, revised, and approved the submitted version.

## The Canine Spinal Cord Injury Consortium (CANSORT SCI)

- Sarah A. Moore, Department of Veterinary Clinical Sciences, The Ohio State University College of Veterinary Medicine, Columbus, OH, United States
- Natasha J. Olby, Department of Clinical Sciences, North Carolina State University College of Veterinary Medicine, Raleigh, NC, United States
- Jonathan M. Levine, Department of Small Animal Clinical Sciences, College of Veterinary Medicine and Biomedical Sciences, Texas A&M University, College Station, TX, United States
- Melissa J. Lewis, Department of Veterinary Clinical Sciences, Purdue University College of Veterinary Medicine, West Lafayette, IN, United States
- Nick D. Jeffery, College of Veterinary Medicine, Texas A&M University, College Station, TX, United States
- Ronaldo Casimiro da Costa, Department of Veterinary Clinical Sciences, College of Veterinary Medicine, The Ohio State University, Columbus, OH, United States
- Yvette S. Nout-Lomas, Department of Clinical Sciences, Colorado State University, Fort Collins, CO, United States
- Joe Fenn, Department of Clinical Science and Services, Royal Veterinary College, Hatfield, United Kingdom
- Nicolas Granger, The Royal Veterinary College, University of London, Hatfield, United Kingdom; CVS referrals, Bristol Veterinary Specialists at Highcroft, Bristol, United Kingdom
- Ingo Spitzbarth, Faculty of Veterinary Medicine, Institute of Veterinary Pathology, Leipzig University, Leipzig, Germany
- Veronika M. Stein, Division of Clinical Neurology, Department for Clinical Veterinary Medicine, Vetsuisse Faculty, University of Bern, Bern, Switzerland
- Andrea Tipold, Department of Small Animal Medicine and Surgery, University of Veterinary Medicine Hannover, Hannover, Germany
- Ji-Hey Lim, Department of Veterinary Medicine and Surgery, MU Veterinary Health Center, University of Missouri, Columbia, MO, United States
- Holger Volk, Department of Small Animal Medicine and Surgery, University of Veterinary Medicine Hannover, Hannover, Germany.

## Conflict of Interest

The authors declare that the research was conducted in the absence of any commercial or financial relationships that could be construed as a potential conflict of interest.

## Supplementary Material

Supplementary Video 1 Assessment of deep pain perception in an ~3-year-old male castrated mix breed dog. He is paraplegic at time of testing. Pressure is applied over the lateral and medial digits of the left hind and there is a withdrawal reflex but no behavioral acknowledgment of the stimulus. However, when pressure is applied to digits of the right foot the dog withdraws the limb and rapidly sits up and looks around, indicating that he has sensation in this foot.
